# Mentalizing in schizophrenia is more than just solving theory of mind tasks

**DOI:** 10.3389/fpsyg.2013.00083

**Published:** 2013-02-27

**Authors:** Giancarlo Dimaggio, Raffaele Popolo, Giampaolo Salvatore, Paul H. Lysaker

**Affiliations:** ^1^Centro di Terapia Metacognitiva InterpersonaleRome, Italy; ^2^Department of Psychiatry, Roudebush VA Medical Center, Indiana University School of MedicineIndianapolis, IN, USA

Scherzer et al. (2012) offer an important step forward in the literature by exploring the relationship between a range of different measures of Theory of Mind (ToM) with a small sample of patients with schizophrenia experiencing significant symptomatology. Consistent with earlier work (Brüne, 2005; Bell et al., 2010), they note that impairments in the ability to make sense of the mental states of others involves multiple components that cannot be reduced to one another. To study this they adopt a wide battery of five tasks ranging from identifying: emotions in photographs of eyes, characters intentions within stories, faux-pas, and intentions on the basis of simple hints. They also use what they define as a real-world task, which is attributing mental states to characters be seen in short movies who are undergoing complex interactions. All the tasks discriminated well between clinical and community participants and were relatively closely related to one another with the expectation of the eyes recognition test. The authors conclude that results support the possibility that ToM involves two dimensions: “first and second order inferences or beliefs, interpretation of intentions, interpretation of affect and on the basis of the social cognitive ToM content: faux pas, interpreting indirect messages, lies, irony etc., and contexts” (p. 9). They point to the importance of future research which considers the role of content and context of social cognition.

When trying to make sense of these findings within emerging models of dysfunction, we were struck with five issues which remain to be addressed by future work. The first concerns the nature and onset the social cognitive deficits. Presumably these are deficits which could emerge in different ways and at different times in response to different causal factors. The loss of neurocognitive ability, stigma and the collapse of connections with others, preexisting trauma (Lysaker et al., 2011a) and poverty of early attachment have all been suggested separately to contribute to difficulties recognizing and reasoning about mental states but little work has considered whether social cognitive profiles in schizophrenia can be dissociated on the basis of the causes of and timing of when deficits emerged and not just the manifest nature of those deficits.

A second issue concerns the possibility that social cognitive acts also differ from one another in terms of the degree to which they involve making a discrete judgment about phenomenon which are correctly or incorrectly identified (e.g., judging a facial expression) vs. synthesizing information into a larger representation where complexity and coherence are more important than just being right or wrong (e.g., constructing a personal narrative). As we have suggested elsewhere (Lysaker et al., 2013), correctly judging discrete phenomena such as one's performance on a task or the likely intention of someone in a story are certainly relevant, however these acts differ conceptually from metacognitive acts in which persons synthesize a range of information into a complex meta-representation which cannot be said to be right or wrong but which could be to varying degrees rich and flexible and person-specific (Ciaramelli et al., 2013). Discrete and synthetic acts should inform one another and both are necessary for responding effectively to evolving social exchanges. However, different problems would be expected to emerge if one but not the other were more impaired. For instance, to sustain a truly intimate relationship over time it is important to get a decent idea of the specific emotion another is feeling in the moment while also updating a large picture of that person's core identity.

A third concern is that test batteries such as those adopted by Scherzer et al. neglect the reality that understanding mental states is of uttermost importance when thinking about personally relevant matters. If one has been abused for example, when encountering another person the main issue is not just identifying a hint about what to buy at a store but deciding whether the other person should be perceived as a potential perpetrator (Lysaker et al., 2011a) or a source of danger (Salvatore et al., 2012). In this example there may be specific mentalizing difficulties when intense affect has been reactivated by memories of abuse which then create a bias for the interpretation of evil intentions, again something that cannot be measured with the traditional ToM tasks.

A fourth issue concerns the examination by Scherzer et al. of participants exclusively with moderately severe symptoms. Symptom remission appears to be more the rule than the exception (Silverstein and Bellack, 2008) and hence it is unclear whether the participants here were more treatment resistant, not offered state of the art psychosocial treatment or just in a particularly distressed period of their lives. Symptom remission represents only one domain of recovery and has been suggested by multiple studies to be relatively uncorrelated with more subjective domains of recovery including quality of life. Thus to truly understand the extent to which different social cognitive processes can be dissociated persons in a non-acute phases of illness should be considered.

A final issue we would raise concerns the need to study social cognition within treatment. In psychotherapy persons naturally discuss their goals and challenges and are in a position to examine the attributions they make about the mental processes of others (Lysaker et al., 2011b, 2013; Lysaker, in press). We contend that discourse analysis of such interactions will offer unique opportunities to see how persons not only perceive others but then also how they reason about those perceptions. These studies could well assist in the development of such interventions while also deepening our awareness of how mentalistic deficits challenge recovery in real time.

In summary we have suggested that for the literature to move forward and better understand deficits in social cognition in schizophrenia, work is needed which consider: the causal factors and emergence of social cognitive deficits, the extent to which discrete vs. synthetic ideas about oneself and others are at issue, the impact of affective states, the phase of illness in which these deficits occur and how these deficits are manifest during and respond to treatment.
